# Society and its leading role in the history of TB in Brazil: Celebrating 18 years of STOP-TB Brazil

**DOI:** 10.1590/1518-8345.0000.3870

**Published:** 2023-01-06

**Authors:** Liandro Lindner, Marcia Leão, Ricardo Alexandre Arcêncio

**Affiliations:** 1 Brazilian Partnership Against TB/STOP-TB Brazil.; 2 Universidade de São Paulo, Escola de Enfermagem de Ribeirão Preto, PAHO/WHO Collaborating Centre for Nursing Research Development, Ribeirão Preto, SP, Brazil.

**Figure:**
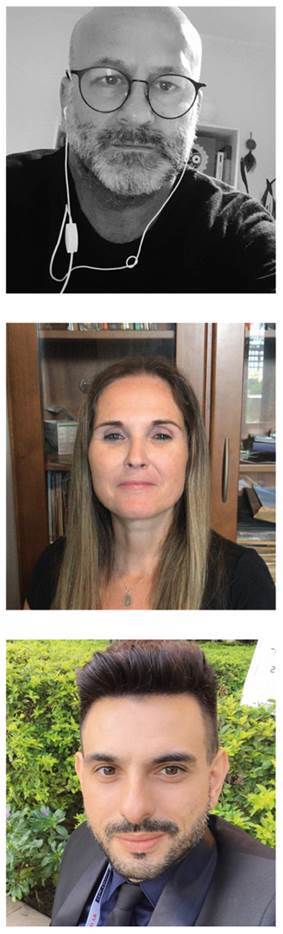

Social Participation (SP) is one of the prerogatives of the Unified Health System (*Sistema Único de Saúde*, SUS) and represents one of the constitutional principles; therefore, in addition to a right, it is a duty of any and all Brazilian citizens^1^. This Editorial aims at highlighting the importance of Civil Society (CS) as a strategic agent for the recognition of the right of people affected by the tuberculosis (TB) to a dignified and good quality treatment, without prejudice, as well as to elucidate the advances of the Brazilian Partnership Against TB in its 18 years of existence, celebrated this year.

TB is a neglected disease and has posed a major challenge to Brazilian public health, as it mainly affects populations in situations of social vulnerability (street people, those in the prison system and indigenous people, among others) who face difficulties accessing health services. Civil Society can play a crucial role with health services and managers, striving to improve the quality of life and dignity of the person, family and communities affected by the disease^1^.

Unfortunately, only 71% of the individuals who initiate the TB treatment in Brazil finish it, when the expectation is a minimum of 85%, therefore remaining behind the World Health Organization (WHO) goals^2^. Such situation can be due to the difficulty this population group finds in following the treatment, which is long, to the side effects of the medications, to the indirect costs to the treatment, to stigma and to preconception about the disease. When a person with TB abandons the treatment, the chance for resistant forms of the mycobacteria increases, whose necessary treatment will be longer (more than 12 months), with more serious side effects and higher costs both for families and for the health system, in addition to the fact that everyone in the community is at risk of contracting the most resistant forms of the mycobacteria. A study showed that 55% of the TB strains are resistant to antibiotics due to treatment interruptions, which requires new approaches to health services, systematic consultations, exams and strategic forms of treatment monitoring and follow-up, person-centered care^3^.

Considering that Brazil is a signatory to the 2030 Agenda Sustainable Development Goals that Target 3.3 recommends elimination of the TB epidemic (among other diseases) by 2030, without suffering and prejudice, its achievement also depends on the restructuring of services and on the mobilization of CS by itself.

In the history on SUS construction and in the field of communicable diseases, CS participation in the struggle for the rights of people with AIDS/HIV was highlighted. This participation culminated in a more dignified treatment in the context of health services, reduction of discrimination and stigma on the part of health professionals and society, and enactment of laws that gave social support, such as pensions, tax exemptions and social benefits to populations living with HIV^4^.

Inspired by this movement to fight for rights, in the TB context, the STOP-TB Brazil campaign, which is the Brazilian Partnership Against Tuberculosis, was launched 18 years ago based on the international guidelines of the Global Plan to stop tuberculosis of the World Health Organization “STOP TB Partnership”. The main objective of these guidelines was to strengthen and accelerate political and social actions to curb the intense spread of TB in the world, when the countries most affected by the disease signed the Amsterdam Declaration^5^ in 2002, during the Ministerial Conference on TB and Social Development, to which Brazil was also a signatory.

In this Declaration, there was an explicit commitment by the countries to monitor and evaluate their National TB Programs (NTBPs), in line with the standards established by the WHO, as well as to support partnerships with non-governmental organizations (NGOs) and the community^5^. This initiative led Brazil, in 2003, to put TB on the health agenda and to agree on strengthening the Directly Observed Short-Term Treatment (DOTS) strategy with the other management spheres as the main instrument to achieve the international goals of TB control^6^.

Potential partners were invited to comprise the Brazilian Partnership, such as the federal government, states, municipalities and civil society, with strong actions to attain the goals. It is worth noting that the Tuberculosis Control Program of the State of Rio de Janeiro and the Tuberculosis Division of the São Paulo State Health Department adopted very peculiar strategies to mobilize civil society to include this community component in the fight against TB. 

In 2003, the Forum of NGOs to Fight Tuberculosis in the State of Rio de Janeiro (FTB-RJ) was created, which played an important role in disseminating information on TB at the local and national levels. The NTBP engaged in comprehensive work to identify and sensitize regional and national institutions to comprise the Partnership.

The Project allowed implementing strategic actions at the local level, resulting in an improvement in the DOTS coverage and a consequent reduction of TB incidence, prevalence and mortality. In order to improve implementation of this Project, the Metropolitan Committees (CAMS) were created, collegiate bodies comprised by representatives from the government and CS and of an advisory and purposeful nature for the discussion of aspects related to the planning, monitoring and evaluation of the Project activities, according to the reality of each metropolitan region.

In the consolidation of the activism segment, the Brazilian Partnership began to occupy important spaces on the national scene, with the launch of the First Joint Parliamentary Front to Fight Tuberculosis and AIDS and the holding of the Third STOP TB World Partner Forum.

The Partnership actions are developed in the field of public policies, executing advocacy actions together with the Legislative and Executive powers at the national and international levels, as well as in the municipalities and states. The Partnership has favored social control through participation in the Rights Councils and in the Public Policy Managing Councils.

Since 2018, the Brazilian Partnership has participated in writing of *Relatório Luz* (Light Report), monitoring and evaluating the country’s progress towards the 2030 Agenda^5^. It also works with the services towards reducing the stigma, as well as for a more dignified treatment for people with TB. It works together with the TB/HIV parliamentary front with stimuli to the proposal of bills that can sustain the social benefits to people with TB, given that 80% of the diagnosed individuals require some kind of social support or assistance.

The Partnership is also a protagonist in the dialog with governments, Funding Agencies and Universities, in the vindication of research and technological innovation, especially of new medications, in an attempt to reduce treatment time and fewer side effects, with a view to the quality of life of people with TB. Breaking drug patents is also an agenda space for their performance.

Thus, the partnership reasserts its commitment to changing the reality experienced by people with TB and their families. To this end, it has brought TB to the political scenario and health agenda, thus collaborating to reducing inequalities as well as ensuring equality and the right of access to health and dignified treatment of populations affected by TB, in the context of the health services.
